# Report on the Origin and Spread of Typhoid Fever in U.S. Military Camps during the Spanish War of 1898

**Published:** 1905-09

**Authors:** 


**Report on the Origin and Spread of Typhoid Fever in U.S. Military Camps during the Spanish War of 1898.** 2 volumes. Washington: Government Printing Office. 1904.

This monumental work has been prepared in accordance with Act of Congress, and is largely due to the interest and appreciation of the late Honourable Secretary of War, Mr. Root. Professor Victor C. Vaughan, Major and Division Surgeon, U.S. Volunteers, has laboured unceasingly to put the full report in such shape as to make it a classic not only for the study of the epidemiologist, but also for the use of the entire medical profession. An abstract of the report was published in 1900. The original board consisted of three medical officers, two of whom have since died—Major Walter Reed, U.S. Army, and Major Edward O. Shakespeare, U.S. Volunteers, whose work receives the highest recognition from the surviving author of these volumes. It was Shakespeare's great work to demonstrate by a detailed study, believed to be without a parallel in the annals of epidemiology, that typhoid fever is a contagious as well as an infectious disease, and that isolation and disinfection are essential procedures in abating certain epidemics of this disease in armies.

